# Dermal absorption of a dilute aqueous solution of malathion

**DOI:** 10.4103/0974-2700.43182

**Published:** 2008

**Authors:** John E Scharf, Giffe T Johnson, Stephen Casey Harbison, James D McCluskey, Raymond D Harbison

**Affiliations:** Department of Environmental and Occupational Health, Center for Environmental/Occupational Risk Analysis and Management, College of Public Health, University of South Florida, Tampa, Florida 33612, USA

**Keywords:** Dermal absorption, malaoxon, malathion, organophosphates, pesticides

## Abstract

Malathion is an organophosphate pesticide commonly used on field crops, fruit trees, livestock, agriculture, and for mosquito and medfly control. Aerial applications can result in solubilized malathion in swimming pools and other recreational waters that may come into contact with human skin. To evaluate the human skin absorption of malathion for the assessment of risk associated with human exposures to aqueous solutions, human volunteers were selected and exposed to aqueous solutions of malathion. Participants submerged their arms and hands in twenty liters of dilute malathion solution in either a stagnant or stirred state. The “disappearance method” was applied by measuring malathion concentrations in the water before and after human exposure for various periods of time. No measurable skin absorption was detected in 42% of the participants; the remaining 58% of participants measured minimal absorbed doses of malathion. Analyzing these results through the Hazard Index model for recreational swimmer and bather exposure levels typically measured in contaminated swimming pools and surface waters after bait application indicated that these exposures are an order of magnitude less than a minimal dose known to result in a measurable change in acetylcholinesterase activity. It is concluded that exposure to aqueous malathion in recreational waters following aerial bait applications is not appreciably absorbed, does not result in an effective dose, and therefore is not a public health hazard.

## INTRODUCTION

Malathion exhibits low mammalian toxicity despite having strong insecticidal properties. Malathion itself has little or no anticholinesterase activity, but as in many other organophosphates, it is bioactivated by mono-oxygenases to produce the potent anticholinesterase malaoxon. After conversion of malathion to its oxygen analog, malaoxon, the active site of acetylcholinesterase (AChE) is phosphorylated, and thereby inhibited by dimethyl or methyl phosphate. Inactivation of AChE causes acetylcholine to accumulate at the neuroreceptor transmission site, resulting in massive overstimulation of the cholinergic system. In humans, malathion and malaoxon are rapidly detoxified by carboxylesterase; the deficient carboxylesterase detoxification pathway in insects is the basis of malathion's insecticidal action.[1]

Most exposures to malathion are through dermal contact. Skin absorption can occur when dermal contact is made during handling and application of the pesticide, when contact is made with chemical residues on plants, fruits, and foliage, in soil, and in dust particles after spraying. A potential non-occupational exposure, however, is from aerial pesticide applications that result in solubilized malathion in swimming pools and other recreational waters humans may come into contact with.[2]

The transference of aerial malathion from the air into a one meter deep body of water typically yields a 22 ppb dilute aqueous solution. Certain areas sprayed at regular intervals, may experience concentration peaks and lulls. As a result, large fluctuations in concentration are known to have occurred in “Medfly hot zones”, DC-3 flight pattern overlapping zones, and bodies of water subject to storm-water runoff. Peak concentrations have been found in swimming pools of up to 90 ppb.[2,3]

Malathion, when dissolved in bodies of water, is readily oxidized to malaoxon by a variety of mild oxidizing compounds. It is generally recognized that malathion is readily oxidized to malaoxon by swimming pool chlorine concentrations. For example, malaoxon has greatest persistence when pool water is acidic, and can be stable in oxygen saturated water at acidic pH for up to two weeks. Sunlight shortens malathion and malaoxon half-lives in pools to 3 days. Current data suggest that little accumulation of malathion or malaoxon in swimming pools occurs; however it does indicate that low levels can persist for more than a week.[4–6]

The balance of malathion and malaoxon dose in humans may be influenced by concomitant exposure to exogenous malaoxon. Swimmers may be exposed to malaoxon in addition to malathion as the aerial bait lands in the swimming pool and is oxidized by chlorine to this much more potent active metabolite, which inhibits AChE activity approximately 68 times more effectively than malathion. However, due to differences in the polarity and lipid solubility of malaoxon, the potential for dermal absorption is far less than that of malathion. Despite this lower potential for absorption, exogenous malaoxon absorption should be considered when evaluating the possible health risks from contaminated recreational waters.[7–11]

Dermal uptake of aromatic chemicals from aqueous solutions can be evaluated using the “disappearance method”. This method requires both hands of each subject to be immersed for two hours in a one-liter beaker, and then a comparison is made between the pre- and post-exposure concentrations of the solution. The best available approach for estimating dermal exposure to malathion in a swimming pool is the model utilized for chloroform (CF) and trichloroethylene (TCE) for baths and showers. This type model is specific for dilute aqueous solutions of lipophilic, organic contaminants.[12–15]

By measuring the dermal absorption of malathion concentrations in an aqueous solution, this investigation examines the dermal absorption rate of malathion and the potential health impact of anticholinesterase activity in recreational swimmers exposed to waters contaminated by aerial malathion applications. The results of this study can be applied to the risk assessment of exposure to malathion contaminated surface waters.

## MATERIALS AND METHODS

### Malathion exposure

Twenty volunteers (2 female, 18 male) aged 25-50 years, with weights ranging from 70-100 kg were subjected to limited dermal exposure of hands and forearms of dilute aqueous solutions of malathion in a laboratory setting. Twenty liter tanks were prepared with dilute aqueous solutions of 50-ppb malathion by dissolving 1 mg of laboratory grade malathion (Supelco PS86, Malathion, Neat) into a distilled water bath. Each twenty liter tank prepared was verified to be within ±30% (±15 ppb) of the nominal target concentration 50 ppb. The malathion exposure tank was stagnant, hand-stirred, and then mechanically stirred during three different stirring exposure phases of the protocol. The tanks were at room temperature (21°C) and swimming pool temperature (29°C or 90°F) during two different temperature exposure phases of the protocol as well. Subjects placed both hands and forearms (total body surface area exposed: Fs = 0.11) into the dilute aqueous 50-ppb malathion solution for up to two (2) hours. Malathion solution assays were then made via the “disappearance method” on the tank solution before and after the exposure period. Volunteer exposure time was gradually increased during several different phases of the protocol from 30 minutes to 120 minutes.

### Malathion quantification and skin abosorption analysis

Malathion assays were performed by passing 500 ml of the tank exposure solution through Bakerbond C18 Speedisks (Part Number 8055-06). Speedisks with extracted malathion solute were then shipped in a sealed container to Ecology and Environment, Inc., for completion of the EPA SW-846 assay method, which is sensitive to 0.5 ppb. Malathion was detected and quantified using gas chromatography/mass spectroscopy.

Calculations of chemical flux into skin from the exposure solution are estimated according to Fick's Law as the difference in chemical mass contained in the exposure solution at the beginning and end of the exposure period, divided by the corresponding exposed dermal surface areas.[16,17] Kp-malathion (permeability constant across the skin) [Table 1] was then derived from the actual Fs (fraction of skin in contact with exposure solution) exposure model. To extrapolate the potential absorption from total body/swimming pool exposure, Kp-malathion was applied to an Fs = 1.00 for both adult [Table 2] and child [Table 3] models. The hazard index (HI) values for theoretical three-hour swimming pool exposures were then calculated based upon experimentally derived Dd (skin dosage of malathion absorbed) and Kp-malathion values. The HI model is a worst-case-scenario analysis and assumes the subject volunteer absorbs all the malathion in the exposure tank, 1 mg, or 12 mcg/kg/day. Since the Reference Exposure Level (REL) is 200 mcg/kg/day, Hazard Index = 12/200 or 0.06, health protection is guaranteed since the estimated dosage is an order of magnitude below the relevant REL and HI.

**Table 1:** Parameters for K_P_ = Dd*Mb/(Dc*De*Fs*As)

Dd = skin dosage of malathion absorbed in mcg
Dc = average concentration gradient of malathion from water to skin
De = duration of contact with contaminated water
Fs = fraction of skin in contact with exposure solution = 0.11
Fsadj = adjustment for Maibach's relative skin absorption = 0.0775
Fs = fraction of skin in contact with water in swimming pool = 1.00

**Table 2:** Adult parameters for Ds = Kp*Dc*De*Fs*AS*Sys / Mb

As = total skin surface area = 21345 cm^2^
Mb = body mass = 85 kg
Hi = Ds/(0.2 mg/kg/day)*(85 kg) = Ds/17mg

**Table 3:** Child parameters for DS = Kp*Dc*De*Fs*As*Sys / Mb

As = exposed skin surface area = 12000cm^2^
Mb = body mass = 34.5 kg
Hi = Ds/(0.2 mg/kg/day)* (34.5 kg) = Ds/7 mg

In order to assess the absorption of malaoxon produced by malathion conversion in chlorinated swimming pool water, Kp-malaoxon must be estimated. Bogen's empirical formula for Kp values is based upon molecular weight and oil-water partition coefficient values that can be applied to solublized malaoxon. In this case, owing to the more polar qualities of malaoxon, Kp-malaoxon is estimated to be one-half that of Kp-malathion.[12–15]

## RESULTS

**Figure 1:**
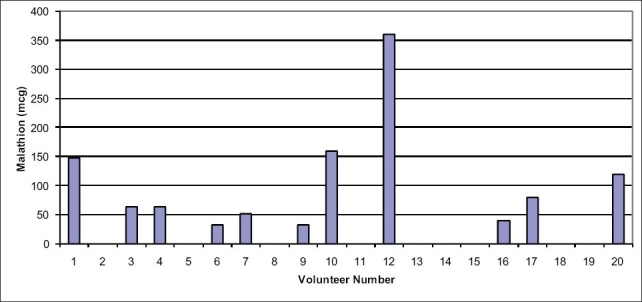
Total dermal absorption by volunteer

**Figure 2:**
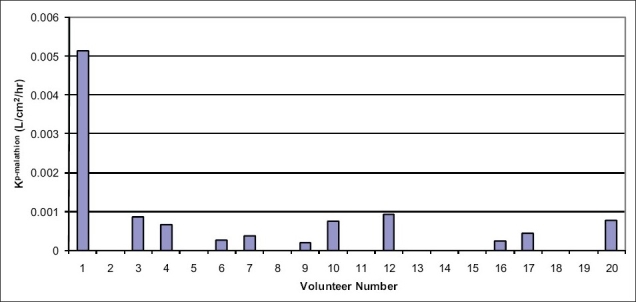
Dermal absorption rate by volunteer

Subject eleven was removed from the study population due to a null concentration of malathion measured during the exposure period, leaving 19 subjects for analysis. Eight of nineteen subjects (42%) absorbed no malathion from solution, receiving no measurable malathion dose during exposure, while eleven subjects (58%) absorbed various amounts of malathion from the aqueous solution [Figure 1]. Experimental Kp-malathion values derived from human volunteer exposures ranged from 0 - 0.0051 L/cm^2^/hr, with a mean value of 0.0005 L/cm^2^/hr [Figure 2].

Using the experimentally-derived Kp-malathion values, the Hazard Index (HI) calculations based on adult swimmers immersed for three hours in 30 ppb malathion ranged from 0-0.06, with a mean HI of 0.01 HI values for child swimmers immersed three hours in 30 ppb malathion ranged from 0-0.08, with a mean HI of 0.01. Applying the experimentally derived Kp-malathion and the estimated Kp-malaoxon, the HI values for adult swimmers immersed for three hours in 30 ppb malathion + 30 ppb malaoxon ranged from 0-2.0, with a mean HI of 0.21. The HI values for child swimmers immersed three hours in 30 ppb malathion + 30 ppb malaoxon ranged from 0-2.8 with a mean HI of 0.29.

## DISCUSSION

As compared to malathion and malaoxon, toluene and chloroform are smaller in molecular weight but with similar oil-water partition coefficients. Thus, according to Bogen's equation, toluene and chloroform should have considerably larger permeability constants (Kp).[12,17] However, the experimentally derived Kp-malathion of .0005 L/cm^2^/hr obtained in this study is actually very similar to those previously obtained by other investigators for Kp-toluene (.0008 L/cm^2^/hr) and Kp-choloroform (0.0001 L/cm^2^/hr).

Utilizing the experimentally-derived Kp-malathion, the HI for adult and child swimmers immersed for three hours in a malathion-only contaminated swimming pool water ranged from 0-0.08. These data indicate that exposure to dilute aqueous malathion solutions following usual aerial bait applications is not appreciably absorbed, and therefore, not a public health hazard.

One consideration with aerial bait application is that malathion is easily oxidized to the more potent malaoxon by chlorine within swimming pools. This is evidenced by the fact that both California and Florida environmental agencies and public health authorities found malaoxon contamination of public and private swimming pools in the days and weeks following aerial bait application.[2,5,15,18,19]

Since malaoxon is 68 times as toxic as malathion, human volunteer exposure is generally considered too dangerous for human experimentation. Therefore, in order to analyze the potential toxicity of external malaoxon contamination and exposure in the swimming pool model, it is necessary to estimate Kp-malaoxon. Since Bogen's empirical formula for Kp values is based upon molecular weight and oil-water partition coefficient values, a value for Kp-malaoxon can be estimated. In this case, owing to the more polar qualities of malaoxon, Kp-malaoxon is conservatively estimated to be one-half that of Kp-malathion.[12,16,17]

The HI for swimmers immersed for three hours in a malathion plus malaoxon contaminated pool was modeled using the experimentally-derived Kp-malathion, and Bogen's technique to estimate Kp-malaoxon. This methodology yielded an average HI of 0.21 for adult swimmers, with a mean HI for child swimmers of 0.29. Although the HI slightly exceeded 1.0 at the upper confidence level (UCL) (two standard deviations) for the malathion plus malaoxon exposure model, the conservative estimate of Kp-malaoxon makes it unreasonable to conclude that the exposure represents any actual public health hazard.

## CONCLUSION

Malathion skin absorption was measured in 58% of the exposed human volunteers by the “disappearance method”. The Kp-malathion was determined to be 0.0005 L/cm^2^/hr. The resulting dose in human volunteers from this controlled exposure to aqueous malathion solution was several orders of magnitude less than the minimal dose known to cause a measurable change in acetylcholinesterase activity. Using the experimentally determined Kp-malathion, a mathematical model was used to evaluate the exposures of swimmers (both adults and children, with and without exogenous malaoxon present) to aqueous malathion concentrations typically detected after aerial bait applications. The resulting HI values were an order of magnitude below that known to result in anticholinesterase toxicity. These data indicate that exposure to aqueous malathion following usual aerial bait applications is not appreciably absorbed, and therefore is not a public health hazard.
